# TMEM207-mediated the impairment of skin regeneration through YAP sequestration in an allergic contact dermatitis model

**DOI:** 10.1016/j.bbrep.2025.102409

**Published:** 2025-12-11

**Authors:** Shusuke Nomura, Yusuke Kito, Chiemi Saigo, Tamotsu Takeuchi

**Affiliations:** Department of Pathology and Translational Research, Gifu University Graduate School of Medicine, Yanagido 1-1, Gifu, 501-1194, Japan

**Keywords:** TMEM207, YAP, Allergic contact dermatitis

## Abstract

**Aim:**

The Yes-associated protein (YAP) family of transcriptional coactivators has emerged as a potent promoter of cell proliferation in many types of stem/progenitor cells and cancers. Skin is a squamous epithelium that is continuously regenerated by stem/progenitor cells in the basal layer and is capable of wound healing, and YAP also plays an important role in maintaining skin homeostasis and cellular proliferation. Therefore, we focused on YAP and investigated the importance of YAP regulation in allergic contact dermatitis from the perspective of wound healing and regeneration.

**Methods:**

We investigated the expression and pathological characteristics of Transmembrane protein 207 (TMEM207), focusing on YAP-mediated regulation in atopic models, as abnormal TMEM207 expression may cause various functional abnormalities or regulate the function of YAP.

**Results:**

TMEM207 was detected not only in the stomach and large intestine but also in the bulge region of the sebaceous glands and hair roots in mice expressing TMEM207. In addition, ATP binding cassette subfamily B member 1 (ABCB1) expression decreased in the sebaceous glands, and MIB E3 Ubiquitin Protein Ligase 1 (MIB-1) expression diminished in the epidermis. Furthermore, although we were unable to confirm the binding of TMEM207 to NEDD4, we confirmed the binding of TMEM207 to the Yes1-associated transcription factor (YAP) by immunoprecipitation, and a decrease in the nuclear localization of YAP was observed by immunohistochemical staining.

**Conclusion:**

Our findings indicate that abnormal expression of TMEM207 is involved in the decline of skin regeneration capacity through YAP, leading to the aggravation of Allergic contact dermatitis.

## Highlights

• TMEM207 was detected in the sebaceous glands and the bulge region of hair follicle in mice expressing TMEM207.
• MIB-1 expression was decreased in the epidermis in mice expressing TMEM207.
• Although TMEM207 does not bind to NEDD4, the binding of TMEM207 to YAP was confirmed by immunoprecipitation.
• Abnormal expression of TMEM207 is involved in the skin regeneration capacity via YAP.

## 1 Introduction

The skin, which covers the entire body, has a “barrier function” that retains moisture, prevents the intrusion of foreign substances, such as harmful microorganisms, and plays an important role in maintaining homeostasis of the body [1]. Normal skin has a stratum corneum on its surface, covered by a sebum film. Sebum is a lipid secreted by the sebaceous glands, and the stratum corneum and sebum prevent the intrusion of irritants and allergens. If the moisture content of the skin or sebaceous glands decreases, the skin barrier function decreases, leading to dryness and the development of dermatitis, such as Allergic contact dermatitis and psoriasis [2]. Allergic contact dermatitis (ACD) is an eczematous disease caused by exposure to an allergen and a delayed type IV hypersensitivity reaction [3]. It affects one in five people and can affect anyone, regardless of age [4]. Allergens come from everyday use in the workplace and in daily life and include ingredients in cosmetics, chemicals, and pharmaceuticals [5]. Symptoms of ACD are diverse, including erythema, papules, vesicles, edema, and pruritus [5]. Patients with ACD often experience long-term, chronic pruritus, which significantly impacts their quality of life [6]. To date, various treatments have been used to treat ACD, including topical medications such as emollients and calcineurin inhibitors, and systemic immunosuppressants such as corticosteroids and methotrexate [7]. However, many drugs are difficult to use long-term due to side effects, making it essential to develop new therapeutic strategies that can effectively treat ACD with fewer side effects.

In this study, a skin barrier breakdown and allergy induction model were created by applying a surfactant and 2,4-Dinitrofluorobenzen(DNFB)to mouse skin, which was subsequently analyzed [8]. The transgenic mice used in this study expressed TMEM207 in their sebaceous glands and the bulge region involved in the skin regeneration of the hair root [9], and we examined the involvement of TMEM207 in skin regeneration and sebaceous gland secretion.

## 2 Materials and methods

### 2.1 Animals

All animal studies were performed in accordance with the ARRIVE (Animal Research: Reporting of In Vivo Experiments) guidelines. All animal experiment protocols were approved by the Animal Care and Use Committee of Gifu University Graduate School (approval no. AG-P-N-20250130; approval date: October 2, 2025; Gifu, Japan). All experiments were performed using 6-8-week-old C57BL/6 mice (22–27 g, Jackson Laboratory Japan, Inc, Shiga, Japan). The process of creating transgenic mice [C57BL/6-Tg (ITF-TMEM207)] is described in the following section. The mice were kept in the SPF animal facility at Gifu University.

### 2.2 Generation of the C57BL/6-Tg (ITF-TMEM207) mouse line

The detailed procedure for generating transgenic mice with a C57BL/6 background and cassette vector has been previously reported [9]. Details of the transgenic mice used in this study have been described previously. The experimental protocol was approved by the Animal Care Committee of the Gifu University Graduate School of Medicine, Gifu City, Japan.

### 2.3 Induction of allergic contact dermatitis

Allergic contact dermatitis was induced as described [10]. DNFB was dissolved in a 4:1 mixture of acetone and olive oil and applied to the ear at a concentration of 0.5 %. The solution was then applied to the ears at a dose of 50 μL for two consecutive days. The solution was then applied again for two consecutive days, 1 and 2 weeks later. Sensitized mice were then sacrificed and analyzed.

### 2.4 Antibodies

Rabbit *anti*-GAPDH antibodies were obtained from Sigma-Aldrich (St. Louis, MO, USA), and rabbit *anti*-ABCB1, *anti*-SCD1, *anti*-NEDD4, *anti*-Ki67, *anti*-Loricrin, and anti-YAP antibodies were purchased from ProteinTech (Proteintech, Inc., USA). A rabbit polyclonal antibody to the synthesized peptide VNYNDQHPNGW (amino acids 40–50 of TMEM207) was generated in our laboratory as previously reported [8].

### 2.5 Immunohistochemical staining and IHC score analysis

Skin tissues were fixed in 10 % buffered formalin and embedded in paraffin. Staining was performed as previously described [11]. Primary antibodies were incubated overnight at 4 °C. The primary antibody used was *anti*-TMEM207, *anti*-ABCB1, *anti*-SCD1, *anti*-Ki-67, *anti*-Loricrin, and anti-YAP overnight at 4 °C. We employed the ImmPRESS Polymerized Reporter Enzyme Staining System (Vector Laboratories Inc., Burlingame, CA) as previously reported [12]. The immunostaining score was based on the Allred score. The proportion of positive cells (proportion score; PS) and staining intensity (intensity score; IS) were determined, and the two were added together to calculate the total score (TS). Nuclear and cytoplasmic staining were scored for each 400x field, and analysis was performed on three fields. Ki67 staining was performed on nuclear expression only, while YAP staining was performed on both nuclear and cytoplasmic expression.

### 2.6 Reverse transcription polymerase chain reaction (RT-PCR) and quantitative real-time PCR

Synthesis of cDNA from total RNA and subsequent PCR was performed using an RT-PCR kit (TaKaRa), as previously described. This instruction was executed based on the manufacturer's instructions. Real-time PCRs were executed using an SYBR Green Reaction Kit according to the manufacturer's instructions (Roche Diagnostics, GmbH, Mannheim, Germany) in a LightCycler (Roche Diagnostics, Mannheim, Germany). The following primers were used for real-time RT-PCR: real-time quantitative PCR primer for mouse *Il-4, 5*- GTCATCCTGCTCTTCTTTCTCG-3 (forward) and 5- CTCTCTGTGGTGTTCTTCGTTG-3 (reverse); mouse *Il-13*, 5- CAGAGGCCATGCAATATCCTC-3 (forward) and 5- CAGCATGGTATGGAGTGTGGA-3 (reverse); mouse *Il-33*, 5- TCCAACTCCAAGATTTCCCCG-3 (forward) and 5- CATGCAGTAGACATGGCAGAA-3 (reverse); mouse *Ccl17*, 5- GAGCTGGTATAAGACCTCAGTGGAG-3 (forward) and 5- TGGCCTTCTTCACATGTTTGTC-3 (reverse); mouse *Ccl11*, 5- CCCAACACACTACTGAAGAGCTACAA-3 (forward) and 5- TTTGCCCAACCTGGTCTTG-3 (reverse); mouse *Gata3*, 5- TCCTCCTCCTCCTCTACGCT-3 (forward) and 5- TGACCACACTGCACACTGAT-3 (reverse); mouse *Stat6*, 5- TGAGCCAGATAACCATGCCC-3 (forward) and 5- CACAGCATGTTCCTGGGACT-3 (reverse); mouse *Post*, 5- TAGCCCAATTAGGCTTGGCATCC-3 (forward) and 5- TAAGAAGGCGTTGGTCCATGCT (reverse); *Gapdh*, 5- TCCTGGTGACTTTGTATATGCGT (forward) and 5- TTCTCGGGCGGGTAATCTTC-3 (reverse); *Tmem207*, 5- GCTTTTCTGTCTTCAGTGCTGGC (forward) and 5- GTTGGACTTCCAGCCATTTCTGC-3 (reverse); *Pparg*, 5- GTACTGTCGGTTTCAGAAGTGCC (forward) and 5- ATCTCCGCCAACAGCTTCTCCT-3 (reverse); *Scd1*, 5- GCAAGCTCTACACCTGCCTCTT (forward) and 5- CGTGCCTTGTAAGTTCTGTGGC-3 (reverse); *Ctgf*, 5- TGCGAAGCTGACCTGGAGGAAA (forward) and 5- CCGCAGAACTTAGCCCTGTATG-3 (reverse); *Cyr61*, 5- GTGAAGTGCGTCCTTGTGGACA (forward) and 5- CTTGACACTGGAGCATCCTGCA-3 (reverse). The DCT values were normalized to *Gapdh* for each triplicate set in both the wild-type (control) and C57BL/6-Tg (ITF-TMEM207) mouse group. The values for the C57BL/6-Tg (ITF-TMEM207) mouse group were then calculated for each target gene as the fold change relative to the mean values for the wild-type group (control; set to 1.0).

Standard deviations were computed for triplicate sets. In addition, Student's t-tests were performed to determine significant differences among groups, with P < 0.05 considered statistically significant.

### 2.7 Western immunoblotting and co-immunoprecipitation (co-IP) assays

Prior to co-IP, mouse tissues were homogenized in lysis buffer (CelLytic M cell lysis reagent or RIPA buffer; Sigma-Aldrich, St Louis, MO, USA) on ice for 30 min. The soluble fraction of their lysates was isolated by centrifugation, and rabbit *anti*-TMEM207, *anti*-NEDD4, and anti-YAP antibodies were prepared. IP was performed using a Capturem™ IP & Co-IP Kit (TaKaRa) or Dynabead Protein G for Immunoprecipitation (Thermo Fisher Scientific, Waltham, MA, USA) according to the instruction manual. Immunoblotting was performed according to the modified method of Towbin et al. (1979), as previously reported [11]. After blocking with bovine serum albumin, the membranes were incubated with *anti*-ABCB1 rabbit polyclonal antibody, *anti*-NEDD4 antibody, and *anti*-TMEM207. The immunoreactivity was detected using a Western BLoT Ultra Sensitive HRP substrate kit (Takara Bio Inc, japan). Immunoblot bands were quantified by densitometry using LI-COR C-DiGit Blot Scanner imaging software (LI-Cor Biosciences, Lincoln, NE, USA) and normalized to GAPDH signal intensity.

### 2.8 Statistical analysis

RT-qPCR and Western immunoblotting experiments were performed in triplicate, and data including RT-qPCR, Intensity of Western immunoblotting, IHC score are presented in graphs with error bars as the mean and standard error of the mean. Comparisons between the wild-type mouse group and the transgenic mouse group were analyzed using unpaired Student's t-test with Microsoft Excel 2021 software (Microsoft Corporation). P < 0.05 was considered to indicate a statistically significant difference.

## 3 Results

### 3.1 Induction of allergic contact dermatitis by repeated application of hapten

A hapten is an incomplete antigen that is not recognized by the immune system itself but becomes complete by binding to a macromolecule, such as a protein; it is a low-molecular-weight compound that induces an immune response [13]. When 2,4-dinitrofluorobenzene (DNFB), a representative hapten, is applied to the skin of a mouse, it binds to proteins during skin penetration and is taken up by antigen-presenting cells, processed, and transported to the lymph nodes, where it activates lymphocytes [14]. Dermatitis did not occur when DNFB was applied for the first time; however, it did when DNFB was applied again several days after sensitization was established. Skin swelling was not observed immediately after DNFB application but gradually appeared and reached a peak 24–48 h later, resembling a delayed-type reaction.

**Fig. 1:**
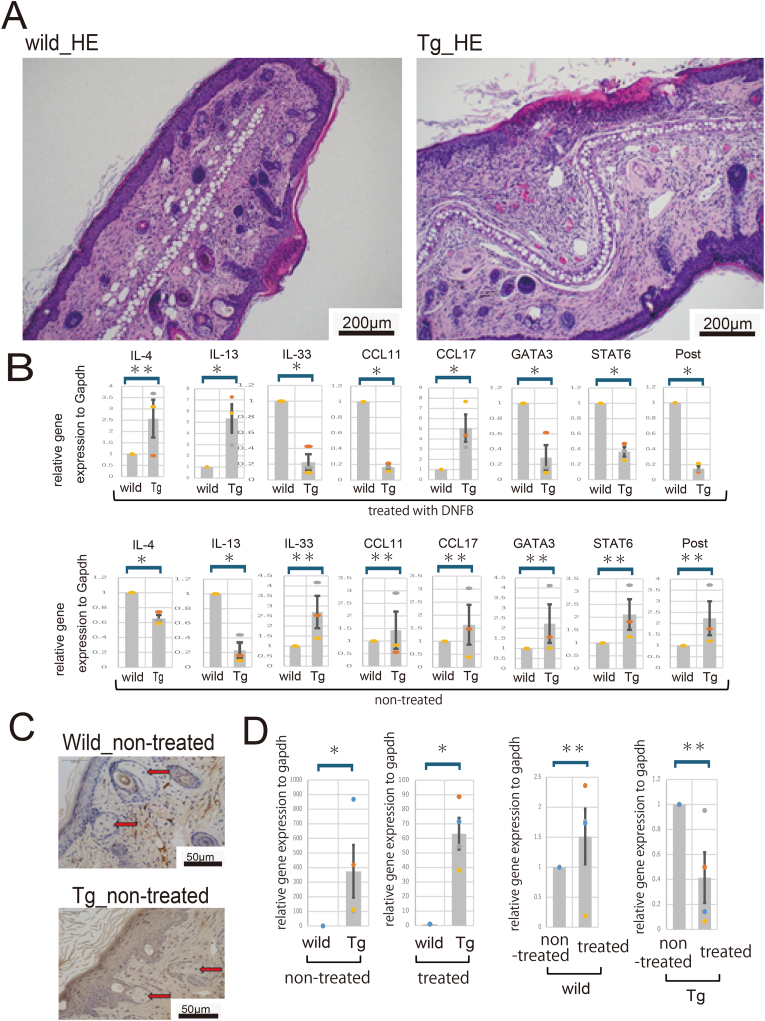
(A): Representative histopathological findings of skin tissues in DNFB-treated C57BL/6-Tg (ITF-TMEM207) mice and DNFB-treated wild-type mice. (B): Comparison of inflammatory cytokines in the skin. In the transgenic mice, Th2-associated cytokines and eosinophil-associated chemokines are elevated. The top row shows mice treated with DNFB. The bottom row shows mice not treated with DNFB. ∗∗ no significant difference; ∗P < 0.05. (C): Representative immunohistochemical staining of the skin using an *anti*-TMEM207 antibody. (D) Comparison of TMEM207 gene expression in skin. High expression of TMEM207 is observed in transgenic mice regardless of DNFB treatment. Furthermore, DNFB treatment did not cause any significant changes in the expression of TMEM207.

Allergic contact dermatitis was induced according to the methods described by Phanuphak [10] and Suzuki et al. [15]. Signs of severe inflammation, such as marked thickening of the epidermis, swelling, and fibrosis of the dermis, and infiltration of eosinophils, lymphocytes, and neutrophils, were observed. Representative histological images of each dermatitis type are shown in (Fig. 1A). Both reflected the pathology of Allergic contact dermatitis, but no significant histological changes were observed.

### 3.2 Comparison of cytokine and chemokine production in skin tissues

We investigated the production of cytokines and chemokines in skin tissue using real-time PCR (Fig. 1B). First, we examined the inflammatory cytokines in wild-type mice and transgenic mice (Tg-mice) that had not treated DNFB. Expression of *Il4* and *Il13* was significantly lower in Tg-mice than in wild-type mice, but no significant differences were observed in other cytokines. Among the cytokines whose levels increased with worsening of Allergic contact dermatitis, a significant increase in the expression of *Il4* and *Il13* was observed. In addition, decreased expression of *Gata3* and *Stat6*, which are transcriptional regulators of Th2 cells, was observed. Among the cytokines *Ccl11*, *Ccl17*, and *Il5*, which are related to eosinophil infiltration, *Ccl17* and *Il5* were significantly upregulated. Furthermore, the expression level of *Periostin (Postn)* decreased, but this was due to short-term exposure; long-term exposure may have produced different results. These findings suggest that overexpression of TMEM207 may have some effect on the production of inflammatory cytokines.

### 3.3 Overexpression of TMEM207 in sebaceous glands of skin tissue

Next, we examined the expression of TMEM207 in skin tissues by immunohistochemistry and found high levels of TMEM207 in the sebaceous glands and hair follicle bulge regions of transgenic mice. Almost no expression was observed in the wild-type mice. Representative tissue images are shown in Fig. 1C.

As previously reported, these transgenic mice contain a foreign gene, TMEM207, inserted into the major satellite repeat sequence at chromosome 2. Expression in the skin was assessed by qPCR and immunostaining (Fig. 1D). It is possible that factors related to the insertion site affect the skin phenotype independently of TMEM207's role in barrier regulation, but this possibility is unlikely because the foreign gene insertion does not disrupt an exon region. Furthermore, DNFB application did not result in the development of skin tumors.

Next, changes in TMEM207 expression were examined using real-time PCR (Fig. 1D). Regardless of DNFB application, there was almost no TMEM207 expression in wild-type mice, while TMEM207 expression was confirmed in Tg-mice. Furthermore, there was no significant change in TMEM207 expression before and after DNFB application in either wild-type or Tg-mice. In other words, DNFB application did not significantly increase TMEM207 expression in wild-type mice, and DNFB application did not significantly increase or decrease TMEM207 expression in Tg-mice either.

### 3.4 TMEM207 expression is not involved in secretory sebaceous glands via ABCB1 in allergic contact dermatitis

**Fig. 2:**
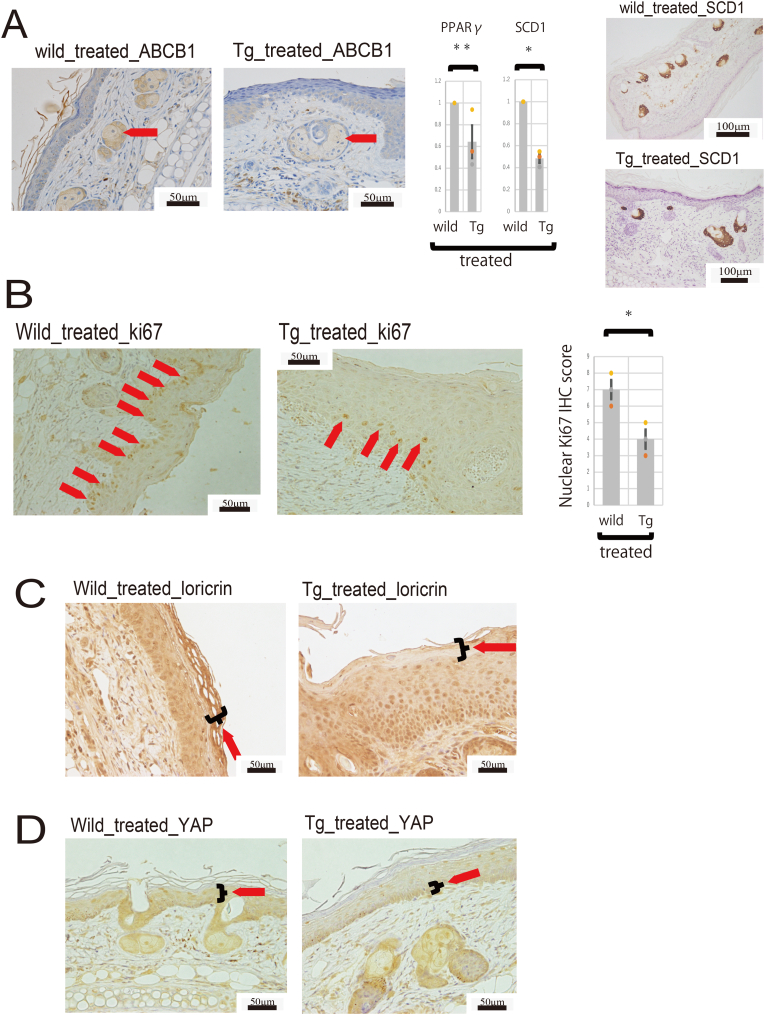
(A): (left) Representative immunohistochemical staining of the skin using an *anti*-ABCB1 antibody. (center) Comparison of PPARg and SCD1 gene expression. (right) Representative immunohistochemical staining of the skin using an *anti*-SCD1 antibody. ∗∗ no significant difference; ∗P < 0.05. (B): Representative immunohistochemical staining of the skin using an *anti*-ki67 antibody and Nuclear Ki67 IHC score. Expression of ki67 was significantly reduced in Tg compared to wild type. ∗P < 0.05. (C): Representative immunohistochemical staining of the skin using an *anti*-Loricrin antibody. (D): Representative immunohistochemical staining of the skin using an anti-YAP antibody.

Sebum secretion in the sebaceous gland involves not only a holocrine mechanism but also an active regulatory one mediated by apoptosis-independent ABC transporters [16]. We investigated how this mechanism was altered in these mice. The results of immunohistochemistry (Fig. 2A) are shown.

When ABCB1 expression in skin tissue was examined by immunohistochemical staining, decreased expression was observed in the sebaceous glands. Sebum secretion volume needed to be examined, so we first evaluated the sebaceous gland differentiation markers PPARg and SCD1 using real-time PCR (Fig. 2A). There was no significant difference in the change in PPARg between the two groups, but SCD1 was significantly reduced in the transgenic mice (Fig. 2A). Therefore, when we examined it using immunohistochemical staining, we observed sufficient expression in the transgenic mice as well, with no major changes (Fig. 2A). Therefore, since only ABCB1 expression showed a significant change in the NEDD4, ABCB1, and sebaceous gland secretion pathway, it seems unlikely that TMEM207 is involved in the sebaceous gland secretion pathway.

On the one hand, ABCB1 is a substrate of NEDD4-1 ligase [16], and it has been reported that it is regulated by NEDD4 [17]. Interestingly, NEDD4 contains a WW domain [18], and TMEM207 may affect the interaction between NEDD4 and ABCB1 by binding to the WW domain of NEDD4 through the PPxY motif [19]. Therefore, we used IP to examine the changes in binding between TMEM207 and NEDD4, as well as those between NEDD4 and ABCB1.

First, we performed IP with TMEM207 and Western blotting for NEDD4, but no binding between TMEM207 and NEDD4 was observed (data not shown). Next, we examined changes in the interaction between NEDD4 and ABCB1. Similarly, we performed IP of NEDD4 and Western blotting for ABCB1, but no decreased interaction between NEDD4 and ABCB1 was observed in C57BL/6-Tg(ITF-TMEM207) mice (data not shown).

### 3.5 TMEM207 partially inhibits the function of YAP but not NEDD4

NEDD4-1-deficient Keratinocytes (KCs) are unable to efficiently activate extracellular signal-regulated kinase 1/2/MAPK and YAP transcriptional coactivators, suggesting that NEDD4-1 plays an essential role in wound repair [20]. Therefore, we performed Ki67 immunostaining to examine the proliferative ability of the skin of these mice (Fig. 2B). In wild-type mice, ki67 expression was relatively localized in the basal layer of the skin, with positive images arranged in a row; however, in the C57BL/6-Tg (ITF-TMEM207) mouse, expression in the basal layer of the epidermis was sparse, suggesting a decrease or change in the proliferation pattern.

Next, ki67 was quantified. The immunostaining score was based on the Allred score. Scores were assigned to each field at 400x magnification, and three fields were analyzed. A significant decrease in the ki67 positivity rate was observed in the transgenic mice (Fig. 2B). Furthermore, to assess whether the skin differentiated normally, we evaluated loricrin expression. The epidermis comprises the basal layer, spinous layer, granular layer, and stratum corneum. The basal layer is crucial for regenerating the epidermis and anchoring it to the basement membrane [21]. As they mature, keratinocytes in the granular layer synthesize envelope proteins such as Loricrin and Filaggrin [22]. Therefore, Loricrin, a principal component of the epidermal keratinocyte envelope, is predominantly expressed in the granular layer during the later stages of epidermal differentiation, serving as a valuable marker of late differentiation. In wild-type mice, loricrin expression extended from the granular layer to the stratum corneum. In contrast, loricrin expression was diminished in the granular layer and stratum corneum of the C57BL/6-Tg (ITF-TMEM207) mouse (Fig. 2C).

**Fig. 3:**
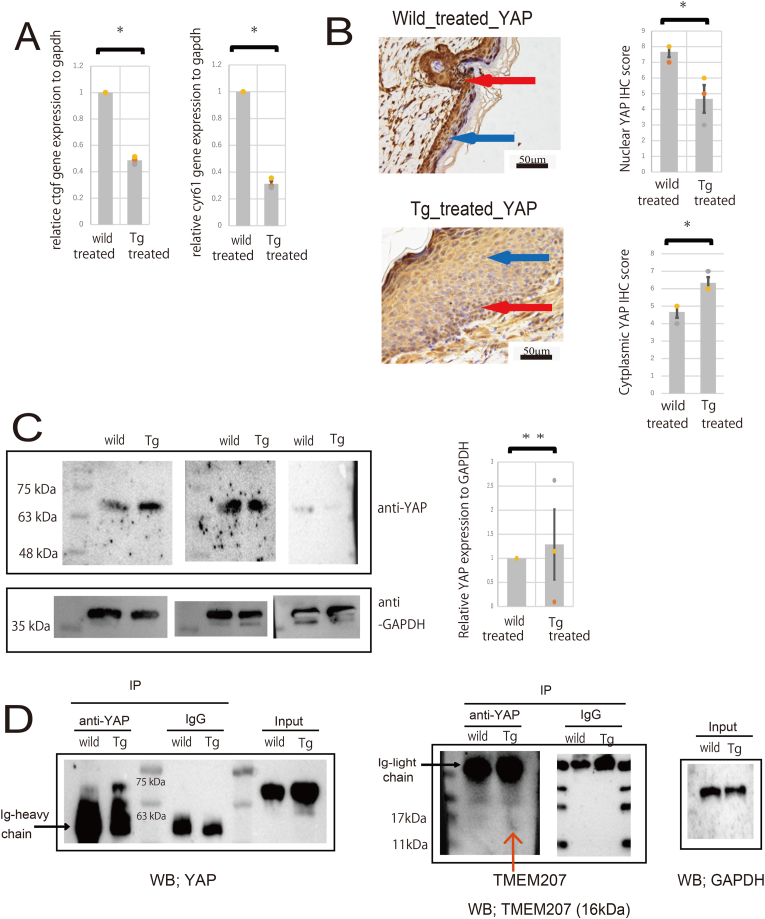
(A) Comparison of downstream target gene analysis (CTGF, CYR61). Significant reductions were observed in Tg mice. ∗P < 0.05. (B) Representative immunohistochemical staining of the skin using an anti-YAP antibody and Quantification of subcellular localization (nuclear vs. cytoplasmic) of YAP immunohistochemical staining. The red arrow indicates nuclear positivity, and the blue arrow indicates cytoplasmic positivity. ∗P < 0.05. (C) Western Blot analysis of YAP in proteins extracted from wild-type and transgenic mouse skin. GAPDH was used as an internal standard. Results from three independent experiments are shown. Measurement of YAP densitometry normalized to GAPDH revealed no significant changes (n = 3; ∗∗n.s = not significant difference, two-tailed unpaired Student's t-test). (D) Wild and Tg skin were co-immunoprecipitated (Co-IP) using an anti-YAP antibody. Western Blotting detected YAP in the precipitate, but not in the IgG isotype control. TMEM207 was also detected in the precipitate from Tg skin. Normal expression of YAP and GAPDH was confirmed in inputs from wild and Tg skin, indicating the presence of these proteins in the cells prior to the co-IP procedure. Representative example of two independent experiments.

Immunostaining was performed for YAP (Fig. 2D). In wild-type mice, YAP expression was observed extending to the surface layer of the epidermis, with pronounced expression in both the nucleus and cytoplasm. Conversely, in the C57BL/6-Tg (ITF-TMEM207) mouse, YAP was predominantly localized to the basal layer, exhibiting reduced expression in the nucleus and cytoplasm. To investigate whether YAP dysfunction actually occurs, we analyzed downstream target genes of YAP (CTGF, CYR61). Analysis using real-time PCR revealed a significant decrease in the expression of downstream target genes of YAP in the transgenic mice (Fig. 3A). YAP-IHC was used to observe and quantitate subcellular localization (nuclear vs. cytoplasmic). As with Ki67, immunostaining scores were based on the Allred score, with a score assigned to each 400x field, and three fields were analyzed. In transgenic mice, nuclear expression was significantly reduced compared to wild-type mice, while cytoplasmic expression was significantly increased (Fig. 3B). These data showing reduced nuclear localization of YAP by immunohistochemical staining suggest YAP inactivation and are thought to be due to inhibition of YAP nuclear translocation.

We therefore confirmed YAP expression in the skin by Western blot, and found no significant difference in expression levels between wild-type and transgenic mice (Fig. 3C). It was therefore suggested that TMEM207 may be inhibiting YAP nuclear translocation.

Notably, YAP contains WW domain [23,24], and it is hypothesized that binding to YAP's WW domain via the PPxY motif of TMEM207 may influence YAP function. Therefore, we explored the interaction between TMEM207 and YAP through IP. Initially, IP was conducted using YAP, followed by Western blot analysis with TMEM207 (Fig. 3D). We observed that TMEM207 interacts with YAP. Thus, it is suggested that TMEM207 binding to YAP hinders its nuclear translocation, potentially leading to functional disruptions and impacting skin regeneration.

## 4 Discussion

Sebum secretion from sebaceous glands and the self-renewal and differentiation of epithelial basal stem/progenitor cell populations are essential for maintaining skin barrier function and homeostasis. Sebaceous glands are accessory skin organs, and their dysfunction and excessive or decreased sebum secretion are closely related to the development of skin diseases [25,26]. Sebum secretion from sebaceous glands has been suggested to involve not only a holocrine mechanism [27] but also an active regulatory mechanism mediated by apoptosis-independent ABC transporters [28,29]. First, we focused on the fact that ABCB1 is a substrate of NEDD4-1 ligase [16], that NEDD4 [17] regulates ABCB1, and that NEDD4 contains three to four WW domains that are responsible for protein-protein interactions [30].

The WW domain interacts with the PPxY motif [19] and is involved in the regulation of ion channels and membrane transporters such as the epithelial sodium channel and α-amino-3-hydroxy-5-methyl-4-isoxazolepropionic acid receptor (AMPARs), growth factor signaling pathways such as Epidermal Growth Factor Receptor (EGFR) and fibroblast growth factor receptor 1 (FGFR1), tumor suppressors such as Beclin 1 and YAP in the Hippo signaling pathway, lysosomal transmembrane proteins such as lysosomal protein transmembrane 5 (LAPTM5), and endocytic regulation proteins [31]. The ubiquitin ligase NEDD4-1 plays an important role in organ development, tissue homeostasis, and cancer. NEDD4-1 knockout mice exhibit impaired keratinocyte proliferation and terminal differentiation, epidermal barrier function, and hair follicle cycling [20], suggesting that it plays an important role in wound repair [20].

In contrast, TMEM207 contains a PPxY motif at its C-terminus that can bind to WW domain proteins, and TMEM207 binds to WWOX via the PPxY motif at its C-terminus [32,33]. Furthermore, NEDD4 and YAP contain WW domains [19,34], and it is speculated that TMEM207's PPxY motif may bind to the WW domains of NEDD4 and YAP, thereby affecting the molecular functions of these proteins that interact with NEDD4 and YAP. In this study, we initially predicted that TMEM207 expression might inhibit NEDD4-mediated regulation. If this were the case, decreased ABCB1 ubiquitination would reduce ABCB1 degradation and increase ABCB1 expression in Tg mice. We then performed a co-IP assay, but failed to demonstrate that NEDD4 binds to TMEM207. This suggests that TMEM207 does not competitively inhibit NEDD4. As a result, although decreased ABCB1 expression was observed, there was no significant change in the expression of sebaceous differentiation markers, so it is unlikely that this affected sebaceous gland secretion. Therefore, it is unlikely that decreased ABCB1 expression led to the worsening of symptoms.

Next, YAP is an essential factor for skin homeostasis and the maintenance of epithelial stem cells [35], which plays a central role in cell regeneration and cancer growth [35].

A recent report showed that epidermal YAP activity induces β-catenin activation and promotes keratinocyte proliferation in mouse skin [36]. The transcription factor YAP is localized in the nucleus of the basal layer of skin, and skin-specific deletion of both YAP and TAZ in adult mice slows basal layer cell proliferation, causes hair loss, and impairs regeneration after wounding [37].

Furthermore, YAP also contains a WW domain [19,34], and we speculated that the PPxY motif of TMEM207 might bind to the WW domain of YAP and affect the molecular function of these proteins that interact with YAP. Therefore, we predicted that TMEM207 expression might inhibit YAP-mediated regulation.

Consequently, the binding of TMEM207 to YAP was demonstrated by IP, and the expression pattern of YAP in the skin was altered, suggesting that this may affect the localization of YAP, that is, inhibit its nuclear translocation. A decrease in the ki67 positivity rate in the skin was confirmed, suggesting a decrease in the proliferative ability, which leads to abnormal YAP function, as has also been shown in mice lacking NEDD4-1. Although overexpressed Tmem207 binds to YAP, the binding between TMEM207 and NEDD4 is unclear, and reduced expression of ABCB1 has no effect on sebaceous gland differentiation or secretion. However, given the reduced ki67 positivity and reduced YAP nuclear translocation, it appears that the main effect is on the pathway involving YAP.

In Conclusions, in Allergic contact dermatitis model mice, expression of TMEM207 in the skin decreased regenerative function of the skin, exacerbating the pathological condition of Allergic contact dermatitis.

## Patient consent for publication

Not applicable.

## CRediT authorship

Contribution statement: Y.K. designed and performed experiments and drafted the manuscript. T. T. conceived the study. C.S. and S.N participated in the experiments. Y.K. and T.T. confirm the authenticity of all the raw data. All authors read and approved the final manuscript.

## Ethics approval and consent to participate

All animal studies were performed in accordance with the ARRIVE (Animal Research: Reporting of In Vivo Experiments) guidelines. The experimental protocol was approved by the Animal Care Committee of Gifu Graduate School of Gifu, Japan (Approval no. 2022-0029).

## Funding

Funding information is not applicable.

## Declaration of competing interest

The authors declare that they have no known competing financial interests or personal relationships that could have appeared to influence the work reported in this paper.

## Data Availability

Data will be made available on request.
