# Supplementary material for: TMEM207-mediated the impairment of skin regeneration through YAP sequestration in an allergic contact dermatitis model
Source: Biochem Biophys Rep. 2025 Dec 11;45:102409. doi: 10.1016/j.bbrep.2025.102409 (PMC12757530; doi:10.1016/j.bbrep.2025.102409)
Supplement: Multimedia component 1 [file mmc1.docx]

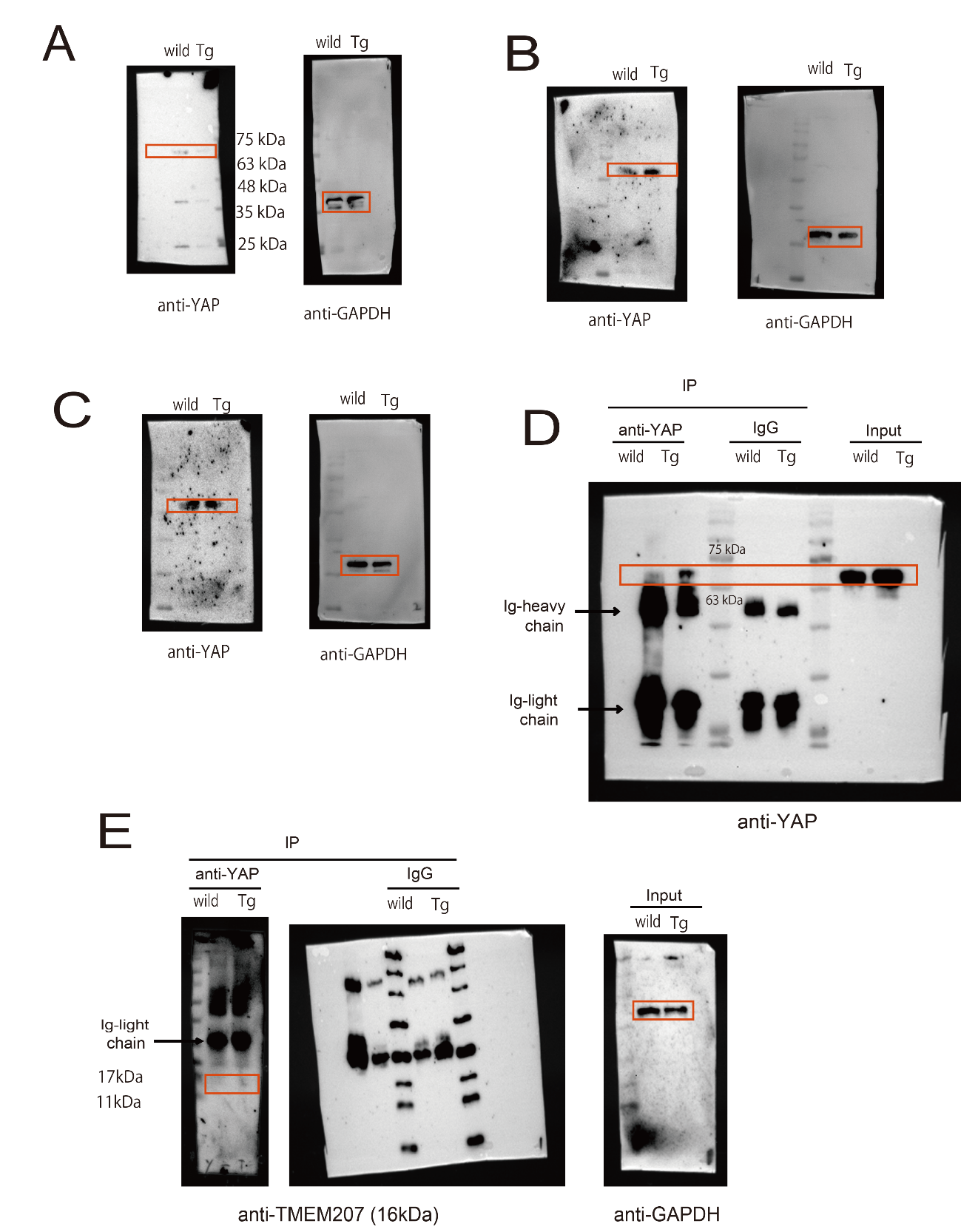


Fig. S1. Full-length, unedited Western blot images.

(A) Full-length, unedited Western blot images corresponding to Fig. 3C.

Blots were sequentially probed for yap (65-78 kDa) and GAPDH (36 kDa), as labeled below the panels. Red boxes indicate the cropped regions displayed in the main figure.1st time.

(B) Full-length, unedited Western blot images corresponding to Fig. 3C. Red boxes indicate the cropped regions displayed in the main figure. 2^nd^ time.

(C) Full-length, unedited Western blot images corresponding to Fig. 3C. Red boxes indicate the cropped regions displayed in the main figure. 3^rd^ time

(D) Full-length, unedited Western blot images corresponding to Fig. 3D.

Blots were sequentially probed for YAP (65-78 kDa) following YAP and IgG co-immunoprecipitation. Red boxes indicate the cropped regions displayed in the main figure.

(E) Full-length, unedited Western blot images corresponding to Fig. 3D.

Blots were sequentially probed for TMEM207 (16 kDa) following YAP and IgG co-immunoprecipitation. Input blot were sequentially probed for GAPDH (36 kDa). Red boxes indicate the cropped regions displayed in the main figure.
